# Identifying Functional Mechanisms of Gene and Protein Regulatory Networks in Response to a Broader Range of Environmental Stresses

**DOI:** 10.1155/2010/408705

**Published:** 2010-04-28

**Authors:** Cheng-Wei Li, Bor-Sen Chen

**Affiliations:** Laboratory of Systems Biology, National Tsing Hua University, Hsinchu 300, Taiwan

## Abstract

Cellular responses to sudden environmental stresses or physiological changes provide living organisms with the opportunity for final survival and further development. Therefore, it is an important topic to understand protective mechanisms against environmental stresses from the viewpoint of gene and protein networks. We propose two coupled nonlinear stochastic dynamic models to reconstruct stress-activated gene and protein regulatory networks via microarray data in response to environmental stresses. According to the reconstructed gene/protein networks, some possible mutual interactions, feedforward and feedback loops are found for accelerating response and filtering noises in these signaling pathways. A bow-tie core network is also identified to coordinate mutual interactions and feedforward loops, feedback inhibitions, feedback activations, and cross talks to cope efficiently with a broader range of environmental stresses with limited proteins and pathways.

## 1. Introduction

Eukaryotic cells have developed protective mechanisms in response to external environmental or physiological changes (stresses). For living organisms, cellular response to sudden environmental or physiological changes determines their fate: life or death. Survivors play an essential role in adjusting the adaptation of the whole organism to such changes or just remain uncorrelated with these changes. When unicellular organisms like *Saccharomyces cerevisiae* suffer from drastic environmental changes, cells may respond swiftly to variations. Therefore, many kinds of signaling pathways exist to construct a protective system to transcriptionally regulate responsible target genes in response to a broader range of environmental stresses. Of these, the high osmolarity glycerol (HOG) pathway, which is the best-understood osmoreponsive system among eukaryotes, is activated by high osmolarity, for example, sorbitol osmotic stress. In contrast to the HOG pathway, the cell-wall integrity pathway is activated by low osmolarity, for example, hypo-osmotic stress. In response to signaling osmotic changes, about 10% of genes are significantly affected in yeast *Saccharomyces cerevisiae* [1–3]. The mitogen-activated protein kinase (MAPK) pathways play an essential role in response to several environmental changes, for example, growth factors, hormones, cytokines and environmental signals, control stress response, cell growth, morphogenesis, and proliferation. Although some nodes of MAPK pathways and the edges of their connections are suggested [4–6], the way they work and connect together in response to specific environmental change is still unclear. Therefore, in this study we try to reconstruct three different directional MAPK signaling pathways (MAPK protein regulatory pathways) in response to the hypo-osmotic stress, the sorbitol osmotic stress, and the pheromone stress, respectively, because pheromone-activated pathways, that is, the pheromone response pathway, share the common components, such as CDC24, STE20, STE50, STE11, STE7, KSS1, and STE12, with the HOG pathway [7, 8]. 

**Figure 1:**
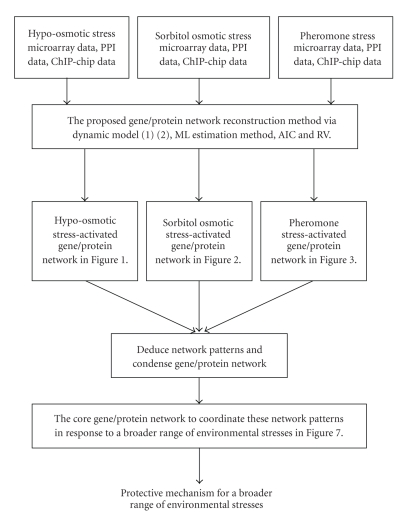
The flowchart of the proposed reconstruction method for gene/protein networks in response to a broader range of environmental stresses. Based on the microarray data, protein/protein interaction data and ChIP-chip data, a gene/protein network is reconstructed in response to each specific stress in Figures 2–4. Then these stress-activated gene/protein networks are condensed to find a bow-tie core gene/protein network in Figure 8 in response to a broader range of environmental stresses.

In recent studies, systems biology method and computational systems biology schemes have been widely used to construct dynamic models for gene regulatory networks [9–14]. In order to reconstruct stress-activated gene and protein regulatory networks (see the flowchart of gene/protein network reconstruction in Figure 1), we develop regulatory dynamic models which can reconstruct not only a directional protein regulatory pathway but also the responsible gene transcription regulatory network, simultaneously. When the time-course data of a dynamic system is available, the use of system dynamic modeling is more appropriate than the use of a statistical approach, because it can model the dynamic behavior of the system. Recently, the dynamic model of genetic network could be identified by microarray data via the so-called reverse engineering methods [15, 16]. By incorporating various types of genomic data, for example, motif information, Chromatin ImmunoPrecipitation on chip (ChIP-chip) data, and microarray data, transcriptional modules have been explored [17, 18]. Previous studies have examined dynamic system identifications of upstream regulators to target genes based on gene expression profiles under cell cycles [19, 20]. However, the binding information of transcription factors (TFs) to promoters of DNA sequences has not been fully utilized to seek for the possible regulations between TFs and target genes. Thus, in this study, microarray data [2, 21], ChIP-chip data [22], and Protein-protein interaction (PPI) data are used to investigate the interplay between internal transcriptional regulations and signaling transduction pathways. Three environmental stresses, that is, the hypo-osmotic stress, the sorbitol osmotic stress, and the pheromone stress (*α* factor), are considered to identify the responsive mechanisms of gene and protein regulatory networks which activate the mostly concerned MAPK pathway in yeast. 

The transcriptional regulatory dynamic model and protein interaction dynamic model are exploited to simultaneously reconstruct the stress-activated gene and protein regulatory networks via a combination of ChIP-chip data, genome-wide microarray data and PPI data (80,537 interacting pairs obtained from BioGRID (the Biological General Repository for Interaction Datasets) (http://www.thebiogrid.org/)). The significant upstream TFs of the target genes and the significant upstream interacting proteins were selected through Akaike Information Criterion (AIC) via backstepping selection procedure in the pruning of identified environmental stress-activated gene and protein regulatory networks [19, 39]. Then we can reconstruct the significant stress-activated signaling pathways and gene regulatory networks under different environmental stresses. After investigating the network patterns of these gene/protein regulatory networks, we can deduce a bow-tie core network to coordinate these network patterns in response to a broader range of environmental stresses (see flowchart in Figure 1). 

Recently, transcription and translation represent structures where common machineries are used to trigger the corresponding biological functions in response to a broader range of environmental stresses, but versatile mechanisms make up the conserved core network. A bow-tie core network previously found for the web network and metabolic network has many inputs and outputs that are connected through a conserved core and versatile weak linkages with extensive signaling pathways governing network responses [40–42]. Various signaling pathways can be interfaced with a core network through versatile interfaces, so that different pathways can be recruited or removed easily without seriously affecting other parts of the network except the network connections in response to the environmental stresses. Therefore, a bow-tie architecture improves the robustness of the network against external perturbations by a robust core where numerous reactions are mediated [43, 44]. In additional to the robustness of the conserved core, a bow-tie architecture also facilitates feedback regulation, because the whole network needs to respond to environmental stresses through a bow-tie core with a minimal effect on other networks [40]. 

Increased stability and switch-like response may exist in a MAPK pathway in response to corresponding stimuli in order to reduce response time [45, 46]. In addition, shutoff mechanism may exist in a MAPK pathway in response to non-corresponding stimuli in order to prevent proliferation of inappropriate pathway activation [47]. In line with the investigations of the network patterns in previous studies [46, 48–51], the patterns can be considered as the specific mechanisms for yeast in response to different environmental changes. Hence, the PPIs of MAPK pathways and transcription regulations are coordinated to make up for the corresponding mechanisms and adapt to environmental changes. Therefore, in the coordinate pathways we are concerned with two feedback loops, that is, feedback activation and feedback inhibition, which provide switch-like response and shutoff mechanism for the networks, respectively. In a MAPK pathway, feedback inhibition, which is used to decrease the protein concentration of the pathway by negative transcription regulation performed by the downstream TF of the pathway, acts like a shutoff mechanism to prevent the proliferation of inappropriate pathway activation [7, 47]; while feedback activation, which is used to increase the protein concentration of the pathway by positive transcription regulation performed by the downstream TF of the pathway, can increase stability and reduce response time to environmental stimuli [45, 46]. Moreover, signal accelerators may exist in a MAPK pathway in order to promote the efficiency of signaling. Since mutual interaction, that is, the action of signaling proteins on each other, is expected to increase reactants, which can dramatically accelerate signaling [52], mutual interactions in gene and protein regulatory networks are concerned with to investigate which stress-activated pathways are likely accelerated by mutual interactions. If enough time profiles of microarray data and PPI data for the reconstruction of gene and protein regulatory networks are available, the proposed method can be applied to investigate some specific mechanisms of gene and protein networks for all species. Finally, the coordination of these specific mechanisms of gene and protein network via a bow-tie architecture will be also investigated in detail to provide us with in depth insight into the nature of this protective system against a broader range of environmental stresses on yeast.

## 2. Gene Transcriptional Regulatory Network

The flowchart of gene/protein network reconstruction algorithm is shown in Figure 1. First, we need to develop a dynamic model for gene/protein network. In general, biological regulatory systems can be described using simple mathematical models to reveal their biological characteristics. Two coupled stochastic differential equations (SDEs) are constructed in the description of the causality of gene and protein regulatory network, respectively [19].

Let *X*_*i*_(*t*) and *Y*_*i*_(*t*), respectively, denote the *i*th gene expression profile and the corresponding protein level at time point *t*. The dynamic mechanisms lying in the transcript level *X*_*i*_(*t*) are formulated by the following dynamic model:

(1) $$\begin{array}{cc} {\dot{X}}_{i}\left(t\right)+a_{i}X_{i}\left(t\right)=\overset{J_{i}}{\underset{j=1}{\sum }}b_{i,j}Y_{j}\left(t\right)+b_{i,0}+\varepsilon_{i}\left(t\right) & \\ \;\text{for}\;\;i=1,2,\ldots ,O, & \end{array}$$

where *Y*_*j*_(*t*) are the protein levels. In (1), three dynamic parameters are used to describe the dynamic interplaying behavior of the transcription regulatory model: (i) degradation role of mRNA expression, *a*_*i*_  (*a*_*i*_ ≥ 0), (ii) transcription regulation of the *i*th gene by the *j* *th* regulatory protein (TF) with kinetic parameter *b*_*i*,*j*_, and (iii) basal level of gene expression of the *i*th gene, *b*_*i*,0_. Additionally, *ε*_*i*_ stands for the measurement noise of microarray data or residue of the dynamic model. *J*_*i*_ is the number of candidate TFs binding to the promoter regions of the *i*th target gene obtained from the ChIP-chip data [22]. We set the *P*-value threshold for.001 to choose the candidate TFs of each target gene among 203 TFs by the ChIP-chip data. Here, the protein level, *Y*_*j*_(*t*), is obtained by the sigmoid function (Hill function) as the translation from mRNA to protein [53, 54], that is, *Y*_*j*_(*t*) = (*X*_*j*_(*t*)^*n*^)/[*p*_*j*_^*n*^ + *X*_*j*_(*t*)^*n*^], where *p*_*j*_ is defined as the mean of *X*_*j*_(*t*).

### 2.1. Protein Regulatory Pathway

The dynamic mechanisms of regulatory proteins lying in protein level *Y*_*i*_(*t*) are formulated by the following dynamic model:

(2) $$\begin{array}{cc} & {\dot{Y}}_{i}\left(t\right)+\lambda_{i}Y_{i}\left(t\right)=g_{i}X_{i}\left(t\right)-\overset{K_{i}}{\underset{k=1}{\sum }}d_{i,k}Y_{i}\left(t\right)Y_{k}\left(t\right)+d_{i,0}+\xi_{i}\left(t\right)\\ & \;\;\;\;\;\;\;\;\;\;\;\;\;\;\;\;\;\text{for}\;\;i=1,2,\ldots ,O. \end{array}$$

In (2), three dynamic parameters are used to describe the dynamic interplaying behavior of the regulatory protein system: (i) degradation rate *λ*_*i*_ of protein. (*λ*_*i*_ ≥ 0), (ii) interaction parameter *d*_*i*,*k*_ between the *i*th protein and its *K*_*i*_ upstream proteins, (iii) the protein production rate *g*_*i*_ from the translation of mRNA *X*_*i*_(*t*) in (1) and the basal level *d*_*i*,0_. Additionally, *ξ*_*i*_ is the measurement noise of microarray data or residue of the dynamic model. *K*_*i*_ is the number of upstream regulatory protein candidates interacting directionally with the *i*th protein which are obtained from BioGRID (the Biological General Repository for Interaction Datasets) (http://www.thebiogrid.org/). In BioGRID, two directions of each protein interacting pair are both considered as interaction candidates in the protein interaction dynamic model. Here, the protein data, *Y*_*i*_(*t*), is also obtained from the mRNA level *X*_*i*_(*t*) through the sigmoid function (Hill function) as translation function [53].

As the assumption of previous studies in the mathematical model of protein interactions [55, 56], protein level will be diminished by association of protein-protein interactions. Although PPIs are not always significant for the change of protein level, the term −*d*_*i*,*k*_ *Y*_*i*_(*t*)*Y*_*k*_(*t*) exists in the general form (2) of the dynamic model. The insignificant interactions *d*_*i*,*k*_ will be further pruned off by the proposed AIC backstepping method (see Methods).

In the reconstructions of stress-activated gene and protein regulatory networks, we initially selected the proteins which have been well considered in MAPK pathways. However, which proteins are selected is not crucial, because the proteins in each MAPK pathway under different environmental stresses are updated specifically based on the results of our dynamic models. 

According to the reconstructed protein regulatory networks (or MAPK pathways) under different stresses, we can suggest which MAPK pathway would be accelerated or wired with the ability to avoid noises under a specific stress via the findings of mutual interactions and feedforward loops. Moreover, according to the reconstructed combinative gene and protein regulatory networks, we can suggest which pathway response is likely to be rapidly and stably activated or which is probably mediated to prevent noises by a specific stress via the findings of feedback activations and feedback inhibitions.

### 2.2. Selection of MAPK Pathway-Related Proteins

**Figure 2:**
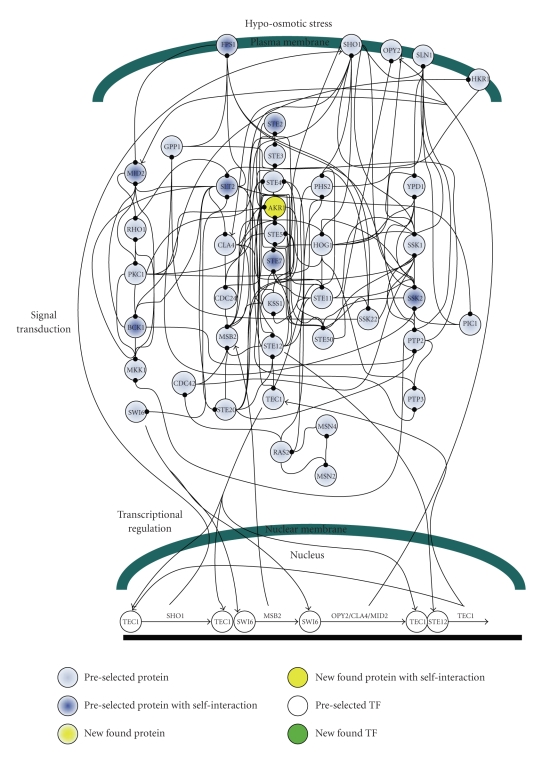
The interplaying between internal gene regulatory network and protein signal pathways in response to hypo-osmotic stress: black-dot stands for protein interactions. The PPIs are shown in Supplementary Table 2.

**Figure 3:**
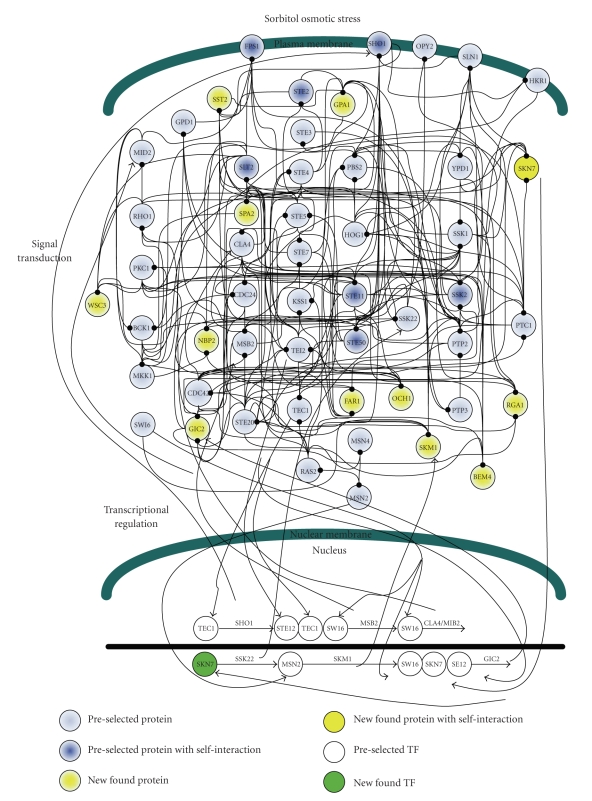
The interplaying between internal gene regulatory network and protein signal pathways in response to sorbitol osmotic stress. The PPIs are shown in Supplementary Table 2.

**Figure 4:**
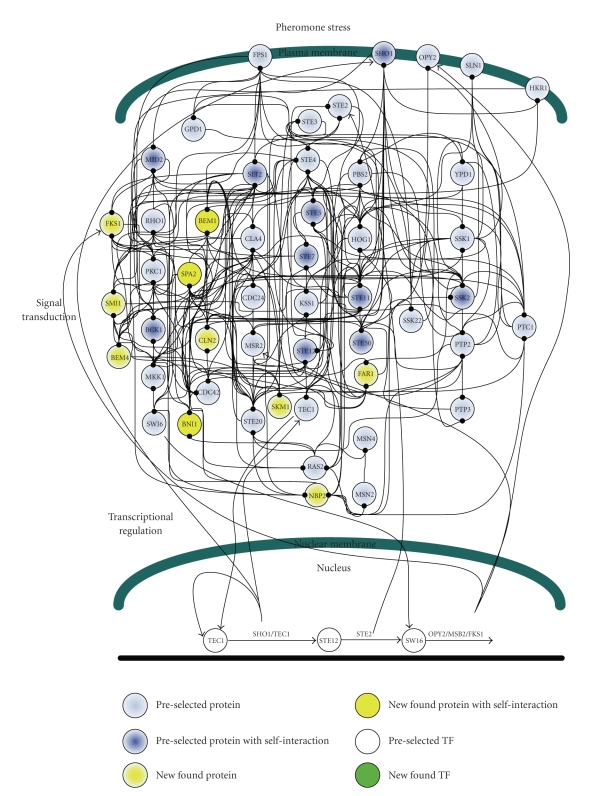
The interplaying between the internal gene regulatory network and outer protein signal pathways in response to pheromone stress. The PPIs are shown in Supplementary Table 2.

In this study, because three environmental stresses, that is, hypo-osmotic stress, sorbitol osmotic stress, and pheromone stress (*α* factor), are investigated either for their oppositely activating stresses or for their highly overlapped signaling proteins, the preselected proteins are selected within three well-concerned MAPK pathways, including the pheromone response pathway, the HOG pathway and the cell-wall integrity pathway [4, 5, 7, 8, 52, 57–60]. The 40 proteins in Figures 2–4 are selected within three MAPK pathways as with the preselected proteins [4–8, 23, 25].

## 3. Results and Discussion

According to ML method combined with the backstepping AIC, the identified models for the interplaying between the gene transcriptional regulatory network and protein regulatory pathway are given as follows:

(3) $$\begin{array}{cc} {\dot{X}}_{i}\left(t\right)+{\hat{a}}_{i}X_{i}\left(t\right) & =\overset{J_{i}^{\prime }}{\underset{j=1}{\sum }}{\hat{b}}_{i,j}Y_{j}\left(t\right)+{\;\hat{b}}_{i,0}+{\hat{\varepsilon }}_{i}\left(t\right)\\ & \text{for}\;\;i=1,2,\ldots ,O,\\ {\dot{Y}}_{i}\left(t\right)+{\hat{\lambda }}_{i}Y_{i}\left(t\right) & ={\hat{g}}_{i}X_{i}\left(t\right)-\overset{K_{i}^{\prime }}{\underset{k=1}{\sum }}{\hat{d}}_{i,k}Y_{i}\left(t\right)Y_{k}\left(t\right)+{\hat{d}}_{i,0}+{\hat{\xi }}_{i}\left(t\right), \end{array}$$

where ${\hat{a}}_{i}$, ${\hat{b}}_{i,j}$, ${\hat{\lambda }}_{i}$, ${\hat{g}}_{i}$ and ${\hat{d}}_{i,k}$ are obtained from the ML parameter estimation method (see Methods). *J*_*i*_ candidate TFs and *K*_*i*_ upstream regulatory proteins are pruned down to *J*_*i*_′ and *K*_*i*_′ by backstepping AIC (see Methods). The covariances of residuals ${\hat{\varepsilon }}_{i}$ and ${\hat{\xi }}_{i}$ are also estimated by the ML parameter estimation method (see Methods). After identifying dynamic regulation function for each target gene and dynamic interaction function for each protein in (3), we could construct the whole gene/protein network by linking the related genes and proteins via the identified transcriptional regulation coefficients ${\hat{b}}_{i,j}$ and protein interaction parameter ${\hat{d}}_{i,k}$ in (3) one by one.

**Figure 5:**
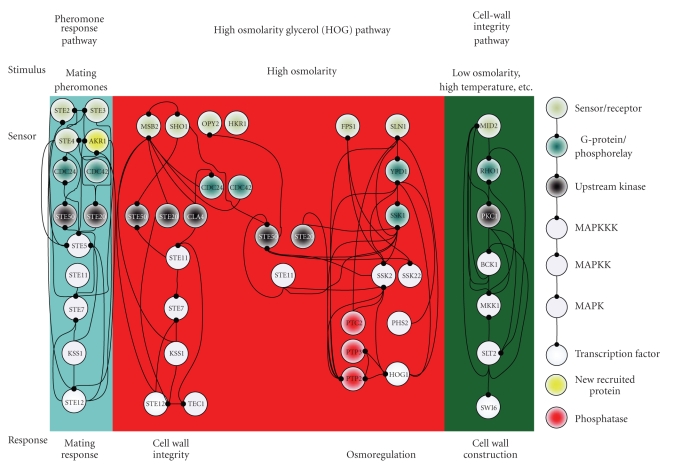
The MAPK module contained in protein regulatory pathways in response to hypo-osmotic stress: the new recruited protein, AKR1, is found and grouped based on Table 1(a). The protein regulatory pathways are the same as those in Figure 2 but with a MAPK module form according to the identified parameter, ${\hat{d}}_{i,k}.$

**Figure 6:**
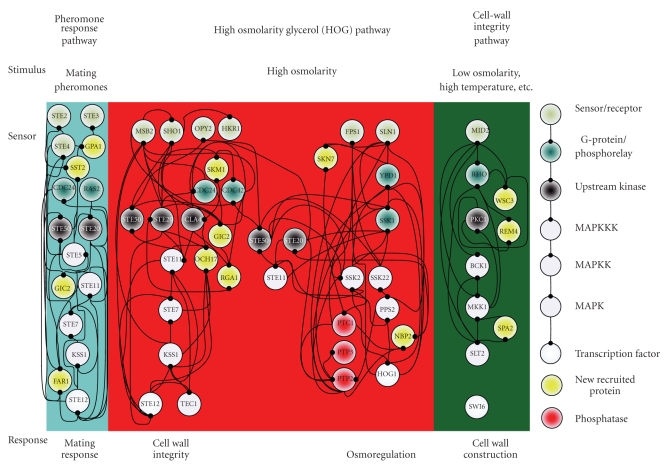
The MAPK module contained in protein regulatory pathways in response to sorbitol osmotic stress: the 12 new recruited proteins are identified and grouped based on Table 1(b). The protein regulatory pathways are the same as those in Figure 3 but with MAPK module form according to the identified parameter, ${\hat{d}}_{i,k}.$

**Figure 7:**
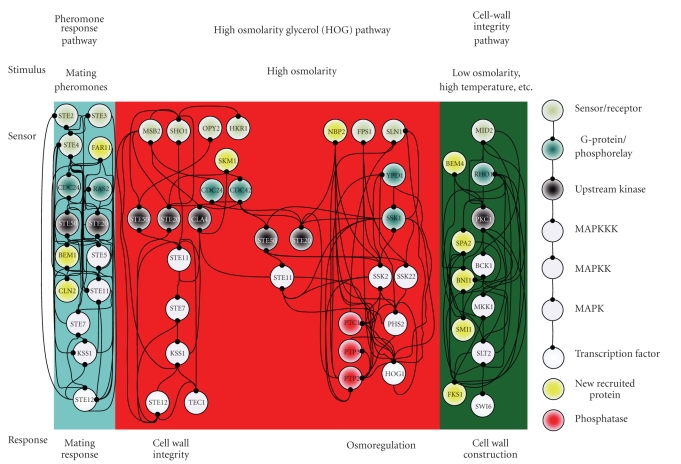
The MAPK module contained in protein regulatory pathways in response to pheromone stress: the 10 new recruited proteins are identified and grouped based on Table 1(c). The protein regulatory pathways are the same as those in Figure 4 but with MAPK module form according to the identified parameter, ${\hat{d}}_{i,k}.$

According to the identified models, the gene and protein regulatory networks within the 40 preselected proteins are reconnected for the three environmental stresses, that is, hypo-osmotic stress (Figure 2), sorbitol osmotic stress (Figure 3), and pheromone stress (*α* factor) (Figure 4) so that the functional mechanisms in response to environmental stresses will be identified more thoroughly. Furthermore, the protein regulatory pathways in Figures 5–7 that contain the MAPK module [7, 8] are, respectively, extracted from the whole protein signal pathways in Figures 2–4. 

Although the gene and protein regulatory networks are reconstructed according to the identified model, some specific target proteins, which are not included in the 40 preselected proteins, may exist in response to specific stresses. In order to improve the accuracy of the reconstructed gene and protein regulatory network and reveal the specific mechanisms of each MAPK pathway in response to each stress, we try to find the specific target proteins or TFs with a significant number of upstream interacting proteins (>5) in the gene and protein regulatory pathways under each stress (Tables 1(a)–1(c)) (full tables combined with *P*-values and gene descriptions are shown in Tables 1(a)–1(c) in Supplementary Material available online at doi:10.1155/2010/408705). For the significant target proteins of the three stresses (Tables 1(a)–1(c)), we find 1, 12 and 10 new proteins respectively, which are not included in the 40 preselected proteins. The new found proteins probably play an important role in coordinating MAPK pathways in response to specific stresses. In accord with the evidence in Table 1, in order to investigate what possible roles the new found proteins play in response to a specific stress, the target proteins are grouped, respectively, into their possible functional pathways (MAPK pathways), so that we can discuss their possible functions in the sequel. After the grouping of the new found proteins into their specific MAPK pathways in response to each stress, the reconstruction of the gene and protein regulatory network in each stress is improved, as shown in Figures 2–4. 

**Figure 8:**
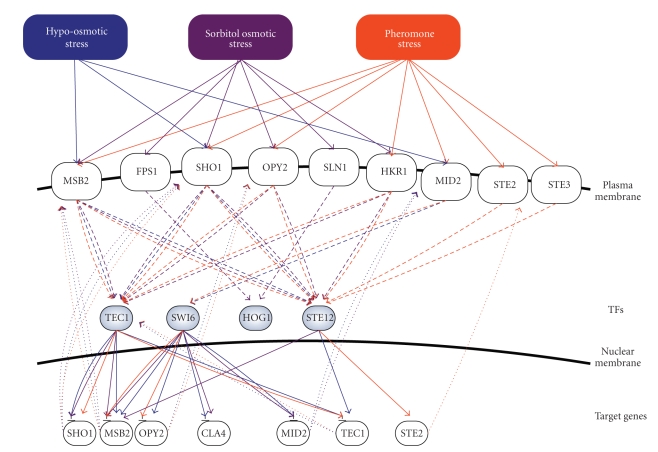
Feedforward activation, feedback inhibition, and activation and cross talks of the condensed gene and protein regulatory networks are coordinated by a bow-tie core, that is, TFs, of gene and protein network in response to hypo-osmotic stress (blue), sorbitol osmotic stress (brown), and pheromone stress (orange), simultaneously: the connections from stresses to TFs are reconstructed according to Figures 5–7. For example, if a MAPK pathway under stress **A**, from membrane protein **B** to the downstream TF **C** of the pathway, is fully connected in Figures 5–7, the relationship, from stress **A** to membrane protein** B** and then to TF **C**, is connected with the same color through the whole pathway. The transcription regulations from TFs to target genes (solid thin lines) are deduced according to Figures 2–4. A target gene transcriptionally regulated under a certain stress may be transcribed to influence the concentration of its protein (TF) (dot lines) in response to the stress. In order to provide the protection against a variety of environmental stresses, the bow-tie core network can detect a broader range of molecular signatures for stresses and invoke effective response under resource-limited condition.

For the convenience of analysis below based on the investigated functional pathways (patterns), the gene and protein regulatory networks in Figures 2–4 are redrawn in a condensed form as shown in Figure 8. There are nine membrane proteins and four TFs in Figure 8 which are the same as those in MAPK pathways (Figures 5–7). The connections in Figure 8 of stresses to target genes are based on Figures 5–7. For example, if a MAPK pathway under stress **A**, from membrane protein (sensor/receptor) **B** to the downstream TF **C** of the pathway, is fully connected in Figures 5–7, the connection, **A**→**B**→**C**, will be drawn in Figure 8. 

Obviously, there exists a bow-tie structure to coordinate these functional pathways (patterns) in the condensed stress-activated network. The bow-tie architecture can entail inherent trade-offs among robustness, resource limitation and performance [41]; that is, the bow-tie core network can enhance the robustness of the stress responsive system by enabling it to cope efficiently with a broader range of environmental stresses with limited resources of proteins and pathways.

According to the coordination of feedback activation, feedback inhibition and cross talk by a bow-tie structure of the condensed stress-activated network in Figure 8, we discuss what kinds of possible mechanisms the stress-activated networks possess to make fast or noiseless responses to a broader range of environmental stresses in the sequel. 

Two MAPK pathways, that is, the HOG pathway, and the cell-wall integrity pathway, activated, respectively, by high and low osmotic stresses are the most concerned MAPK pathways in yeast *Saccharomyces cerevisiae*. Additionally, the HOG pathway shares seven common components with the pheromone response pathway. Therefore, our methods are applied to the three stresses, that is, hypo-osmotic stress, sorbitol osmotic stress, and pheromone stress (*α* factor), to suggest probable specific mechanisms and to deduce their coordinate bow-tie core network in response to a broader range of environmental stresses.

### 3.1. Hypo-Osmotic Stress

When yeast cells are exposed to osmotic stress, several cellular responses, such as solute transporters, solute synthesis, stress resistance, and cell wall structure, are induced by MAPK pathways. Two MAPK pathways, that is, the HOG pathway and the cell integrity pathway (or protein kinase C pathway), have opposing functions stimulated, respectively, by hyperosmotic stress and hypo-osmotic stress. The consistent properties between the cell integrity pathway and the pathway for controlling cell wall metabolism lead to cells with a stronger cell wall at low osmolarity rather than cells at high osmolarity [7, 61]. According to Figure 5 (the hypo-osmotic stress) and Figure 8, we suggest that a strong cell wall of yeast under hypo-osmotic stress may result from two fully connected pathways, that is, the SHO1 branch of the HOG pathway and the cell-wall integrity pathway.

Under hypo-osmotic stress, the cell integrity pathway senses the osmotic changes at the cell wall and controls the production of enzyme involved in cell wall metabolism by transcription regulation to efficiently diminishing turgor pressure [7]. In the cell integrity pathway, we find that 12 genes, which probably exist in the function category of cell walls, that is, PSA1, UTR2, CIS3, GON7, MNN5, CWP1, EXG1, MID2, PAH1, GAS3, FKS3, and KRE6, with *P*-value 4.50E-4 (evaluated by MIPS database) among 121 SWI6 transcriptionally regulating-genes identified by our model, may play an important role in efficiently diminishing turgor pressure. 

Since hypo-osmotic stress accompanied by other environmental changes, which stimulate the pathways mediated by the components just as with the hypo-osmotic stress, exists prevalently, feedforward loops are probably wired to the cell integrity pathway to filter out other signals (noises) [53]. Additionally, protein interactions are expected to increase reactants, which should dramatically accelerate the signaling pathway [52]. According to Figure 5, although the HOG pathway is fully connected except PBS2, and most of the connections are mutually interacted, the SLN1 branch of the HOG pathway is not activated by hypo-osmotic stress. It has been proven that this is due to scaffold proteins, which can provide specificities to the pathway by different bindings and interactions [52, 57, 62]. Therefore, according to Figures 5 and 6, we suggest that the scaffold protein, PBS2 plays an important role in recognizing hypo- and sorbitol osmotic stress in the HOG pathway.

According to Table 1(a), AKR1, which interacts with a significant number (>5) of preselected proteins, is a newly discovered protein. AKR1 has been suggested to play a role as a negative regulator of the pheromone response pathway and is also involved in cell shape control [24]. Therefore, we suggest that AKR1 is an important protein participating in the pheromone response pathway under hyperosmotic stress.

### 3.2. Sorbitol Osmotic Stress

At high osmolarity, two branches of the HOG pathway, that is, the SHO1 branch and the SLN1 branch, are observed to sense osmotic changes and rapidly make internal adjustments. In Figure 6, sorbitol osmotic stress is shown to have many more mutual interactions and feedforward loops in the HOG pathway than hypo-osmotic stress (Figure 5). The connections may make pathways more rapid and more robust (acting against external noise) in response to sorbitol osmotic stress.

According to Table 1(b), the new found proteins are the 12 proteins (highlighted by gray color) which interact with a significant number (>5) of preselected proteins. The 12 proteins are grouped and will be discussed in the following paragraphs based on the research shown in Table 1(b).

Two proteins, namely WSC3, and SPA2, are the new found proteins which are known as members of the cell-wall integrity pathway. WSC3 is involved in the maintenance of cell wall integrity [29], while SPA2 acts as a scaffold protein for MKK1 and MPK1 [7]. In addition, BEM4 is probably involved in the RHO1-mediating signaling pathway [31]. BEM4 is functionally relevant to RHO1 and should play a novel role in the signaling pathway mediated by RHO1. One possible role of BEM4 is to act like chaperone in the stabilizing or folding of RHO1. According to the cell-wall integrity pathway shown in Figure 6, we suggest that RHO1, PKC1 and SLT2 may play important roles in the inactive cell-wall integrity pathway under sorbitol osmotic stress.

In the pheromone response pathway, four proteins, including GPA1, SST2, FAR1, and GIC2, are the new found proteins as shown in Table 1(b). GIC2, whose function is still unknown, can interact with CDC42, and therefore GIC2 is grouped with the pheromone response pathway and the SHO1 branch of the HOG pathway [26]. GPA1, a G_*α*_ subunit, has been involved in mediating pheromone response pathway [7, 27]. SST2 is required to prevent receptor-independent signaling of the pheromone response pathway [28]. Additionally, Far1 is a cell cycle arrest mediator [25].

In the SLN1 branch of the HOG pathway, SKN7, and NBP2 are new found proteins participating with this important pathway under sorbitol osmotic stress. SLN1-YPD1-SKN7 has been proven to act as a phosphorelay system that turns on the HOG pathway until yeast suffers from cell shrinking (Figure 6) [4, 7]. In addition, SKN7 appears to have different functions, such as acting as a transcription factor or a protein in signaling systems, not only mediating different stresses but also linking the cell-wall integrity pathway to the HOG pathway mediated by interacting directly with RHO1 (Figure 3). During yeast adaptation, NBP2 is predicted to act as an adapter, recruiting PTC1 to the PBS2-HOG1 complex in the PTC1 inactivation of HOG1 [23]. We suggest that the activated HOG pathway under sorbitol osmotic stress is due to the unbound NBP2-PBS2 complex which results in HOG1 which cannot be inactivated by PTC1 [23].

In the SHO1 branch of the HOG pathway, the new found proteins OCH1, SKM1, and RGA1 are probably important in response to high osmolarity. The promoter of OCH1, which encodes a mannosyltransferase, responds to the presence of SLN1, and KSS1 is activated by the mutation of OCH1 [7]. Therefore, we suggest that OCH1 participates in the SHO1 branch of the HOG pathway under sorbitol osmotic stress. SKM1, which is similar to STE20 and CLA4, is probably a downstream effector of CDC42, but the function of SKM1 is still unclear [4]. According to [63], CDC42 may promote the phosphorylation of GIC2 by recruiting STE20 and SKM1. Therefore, we suggest that SKM1 is a member of the SHO1 branch of the HOG pathway under sorbitol osmotic stress. RGA1 is suggested as a link between CDC42 and pheromone pathway components [30] (Figure 3). Although the 12 new found proteins are probably important in response to sorbitol osmotic stress, most of them, such as BEM4, GIC2, FAR1, OCH1, SKM1, and RGA1, are functionally unclear, and likely even participate in multiple pathways with complicated roles. We can only infer some possible mechanisms according to previous studies and our results.

### 3.3. Pheromone Stress

When yeast cells are stimulated by mating pheromones, that is, **a**-factor and *α*-factor, haploid cells are matted to a diploid form. Cellular functions, that is, polarized growth, cell cycle arrest in G_1_, cell adhesion, and cell fusion, are activated under the pheromone stress. Therefore, some genes must exist to link up cellular functions and the pheromone response pathway. According to [35], SMI1, suppressor of matrix-association region inhibition, probably coordinates cell wall synthesis and budding [35]. This coordination is also found in our study (Figure 7). Further, it is also found that the three MAPK pathways, that is, the pheromone response pathway, the SHO1 branch of the HOG pathway, and the cell-wall integrity pathway, are probably induced by pheromone stress (Figures 4 and 8). According to Figure 7, mutual interactions apparently exist in two pathways, namely, the pheromone response pathway and the SHO1 branch of the HOG pathway. Therefore, once again we infer that the existence of mutual interactions probably leads to rapid response under environmental stresses. According to [4, 24, 32], because the deletion of AKR1 or BEM1 results in severe effects on cell morphology and viability, and both of them are required for efficient pheromone signaling, in the pheromone response pathway, AKR1 and BEM1 play an important role in cellular morphogenesis. CLN2, a G1 cyclin, is involved in the cell-cycle regulation of MAP kinase signaling [4, 7, 33, 34]. FKS1 and SMI1 are required for the synthesis of a major structural component of cell walls, 1,3-**β**-glucan [4, 7, 35, 36]. BNI1 is known to interact with some members of the Rho-GTPase family and is probably involved in cell integrity signaling [7, 37, 38].

According to the above discussions, we conclude that the SHO1 branch of the HOG pathway under sorbitol osmotic stress (Figure 6) and pheromone response pathway, the SHO1 branch of the HOG pathway, and the cell-wall integrity pathway under pheromone stress (Figure 7) are most likely accelerated by mutual interactions. There are a considerable number of feedforward loops wired to the cell-wall integrity pathway under hypo-osmotic stress as shown in Figure 5, the HOG pathway under sorbitol osmotic stress as in Figure 6, and the pheromone response pathway, the SLN1 branch of the HOG pathway, and the cell-wall integrity pathway under pheromone stress as in Figure 7, to avoid inappropriate stimulations.

### 3.4. Bow-Tie Protective Mechanisms via Coordination of Mutual Interaction, Feedforward and Feedback Loop, and Cross Talks for a Broader Range of Environmental Stresses

The response of yeast to osmotic stress is a transient protection event. According to the observation of [7], the raising time and falling time of HOG1 are, respectively, within about 1 minutes and 30 minutes in response to osmotic shock. An intrinsic feedback mechanism therefore probably exists in the HOG pathway to maintain signaling competence for fast response to osmotic shock and is required for a homeostatic process [5, 64]. According to our results about the intrinsic feedback mechanism, we find that TEC1 and SWI6 are required respectively for feedback inhibition of SHO1 and MID2 under both osmotic stresses, that is, hypo-osmotic stress and sorbitol osmotic stress (Figures 5 and 8). Moreover, at high osmolarity both TEC1 and SWI6 are required, for feedback inhibition of MSB2, while at low osmolarity SWI6 is required for feedback inhibition of CLA4 (Figures 5 and 8). Feedback inhibition acts like a shutoff mechanism to prevent proliferation from inappropriate pathway activation [7]. In addition, feedback activation can increase stability and reduce response time to environmental stimuli [46]. We find that at high osmolarity the SHO1 branch of the HOG pathway rapidly responds to STE12 via STE12-mediated upregulation of MSB2 (Figures 5 and 8), while at low osmolarity the SHO1 branch of HOG pathway rapidly responds to the downstream TF of the cell-wall integrity pathway, that is, SWI6, via SWI6-mediated upregulation of MSB2 and OPY2 (Figures 5 and 8).

Moreover, it is evident that rapid response to the pheromone stresses via feedback activation probably exists in both the pheromone pathway and the SHO1 branch of the HOG pathway respectively via STE12-mediated upregulation of STE2, and via TEC1-mediated upregulation of SHO1 (Figures 7 and 8). Additionally, we found that feedback inhibitions may mediate SWI6-mediated downregulation of both OPY2 and MSB2 in response to pheromone stress (Figures 7 and 8). The feedback regulations implicate that a secondary pathway, that is, the SHO1 branch of the HOG pathway, may be induced by pheromone stress mediated by the cell-wall integrity pathway combined with feedback inhibition to filter out inappropriate signals.

From the results in Supplementary Tables 1(a)–1(c), we find that many TFs are detected in a broader range of environmental stresses, including TEC1, STE12, ADR1, ARR1, CIN5, FKH2, GAT1, and HIR2. It implies that they may be cross talks among the MAPK pathways, such as pheromone response pathway, HOG pathway, and cell-wall integrity pathway. Although these pathways respond to different stresses, they may induce different levels of the same TFs to perform cell protection. In Figure 8, we find that TEC1 and STE12 are detected in hypo-osmotic stress, sorbitol osmotic stress, and pheromone stress. A previous study [4] showed that TEC1 promoter has a regulatory element, FRE (filamentation and invasion responsive element), which is both necessary and sufficient for transcription regulation by upstream activating signals in the pheromone response pathway. Both STE12 and TEC1 regulate the genes containing one copy of a FRE in close proximity to a binding site and are required for the pheromone response. Thus, the FRE of TEC1 promoter provides a positive feedback mechanism for up regulation of TEC1, which can be detected in Figure 4. Furthermore, from the established signaling pathways Gat-Viks and Shamir [65] showed that STE12 and TEC1, which are the TFs in the downstream of the SHO1 branch of the HOG pathway, may respond to osmotic stress. Together, the SHO1 branch of the HOG pathway integrates the need for cell expansion in not only the pheromone stress but also the osmotic stress [7]. Therefore, from Supplementary Tables 1(a)–1(c), we can infer that several TFs may have cross talks in response to a broader range of environmental stresses.

Finally, we conclude that in the cell-wall integrity pathway, RHO1, PKC1, and SLT2 may play an important role in the activation of the cell-wall integrity pathway under both hypo-osmotic stress and pheromone stress. Under hypo-osmotic stress the SHO1 branch of the HOG pathway acts like a secondary pathway, which is accelerated by the activated cell-wall integrity pathway, and both of the pathways, that is, the SHO1 branch of the HOG pathway and the cell-wall integrity pathway, might combine with feedback inhibitions to prevent inappropriate activations (Figures 5 and 8). In the SLN1 branch of the HOG pathway, a scaffold protein, PBS2, can provide the specificity to the activation of the SLN1 branch of the HOG pathway under sorbitol osmotic stress by different kinds of binding and interaction. Under sorbitol osmotic stress, the SHO1 branch of the HOG pathway can itself be accelerated and has the ability to prevent inappropriate activation by feedback inhibition and activation (Figures 6 and 8). In the pheromone response pathway, once again we suggest that a scaffold protein, STE5, can provide the specificity to the activation of the pheromone response pathway under pheromone stress [52, 57, 62]. Therefore, when the pheromone response pathway suffers from pheromone stress via membrane receptors, that is, STE2 and STE3, the activated pheromone response pathway speeds its response by feedback activation via STE12-mediated upregulation of STE2 (Figure 8). The activated pheromone response pathway accelerates itself via feedback activation (Figure 8). Under pheromone stress (*α* factor), the SHO1 branch of the HOG pathway acts like a secondary pathway, that can sense the external signal mediated by the cell-wall integrity pathway, which has the ability to prevent inappropriate activation for the SHO1 branch of the HOG pathway. Additionally, the SHO1 branch of the HOG pathway can also accelerate itself via feedback activation (Figures 7 and 8). Furthermore, we compare the results (Supplementary Tables 1(a)–(c)) with [66] to find the overlap TFs between them. We find that the overlap TFs are participating significantly in not only protein regulatory network but also gene regulatory networks under a specific stress. Under hypo-osmotic stress, sorbitol osmotic stress and pheromone stress, there are, respectively, five TFs, that is, SWI6^1^, PHD1^3^, SWI5^4^, SKN7^6^ and IXR1^9^, five TFs, that is, SKN7^1^, FKH2^3^, HSF1^5^, MBP1^6^, and PHD1^7^, and six TFs, that is, SWI4^2^, PHD1^3^, MBP1^4^, SWI5^5^, FKH2^6^, and SKN7^10^, found in both studies. The superscript of the TFs obtained from of [66, Table 2] shows order of the significance in response to specific stress. Therefore, the other TFs in of [66, Table 1] not found in this study such as INO4^2^ and BAS1^5^ under hypo-osmotic stress, SMP1^2^ and FKH1^4^ under sorbitol osmotic stress and MCM1^1^ under pheromone stress may only play an important role in transcription regulations in response to specific stress. Additionally, SWI6 under sorbitol osmotic stress and HOG1 under both hypo-osmotic stress and pheromone stress are not found in the significant interacting proteins (TFs) by both [66] and our results.

By comparing with previous studies [5–8, 67–69], the false-negative rate is 26.47% (9/34) for high osmolarity glycerol (HOG) pathway under sorbitol osmotic stress and 8.33% (1/12) for pheromone response pathway under pheromone stress (please see Supplementary Table 3). Owing to the insufficient studies in MAPK pathway under hypo-osmotic stress, we can not compare our findings with others under hypo-osmotic stress. 

From the core gene/protein network in Figure 8, feedforward activation, feedback inhibition and activation, and cross talks are coordinated by a bow-tie structure in response to three environmental stresses. In order to provide protection against a variety of environmental stresses, the protection system has to detect a boarder range of molecular signatures for stresses and invoke effective responses. It is clear that this has to be performed under resource-limited conditions because the number of genes, proteins, and pathways that can involve in stress responses is not infinite. Since a bow-tie architecture comprises conserved and efficient core processes with diverse and redundant input and output processes and entails inherent tradeoffs among robustness, fragility, resource limitation, and performance, the bow-tie gene and protein core network has been investigated by the proposed method to coordinate the mutual interactions, feedforward loops, feedback inhibition and activation, and cross talks in response to a boarder range of environmental stresses. 

According to the bow-tie protective network under different stresses in Figure 8, TFs provide as bow-tie cores to trigger appropriate biological responses via receptors (or sensors) in response to environmental stresses. In Figure 8, there exist five loops, which not only trigger the biological response of MAPK pathway but also regulate the expression of membranes proteins (sensors/receptors) via transcription regulations of TFs. For example, the TF, TEC1, triggers the response of cell wall integrity via the receptor (or sensor), SHO1, in response to all three stresses; the TF, TEC1, triggers the response of cell wall integrity via the receptor (or sensor), MSB2, in response to both osmotic stresses; the TF, STE12, triggers the response of cell wall integrity via the receptor (or sensor), MSB2, in response to sorbitol osmotic stress; the TF, STE12, triggers the downstream response of pheromone response pathway via the receptor (or sensor), STE2, in response to pheromone stress; and the TF, SWI6, triggers the response of cell wall construction via the receptor (or sensor), MID2, in response to hypo-osmotic stress. Therefore, these findings in Figure 8 obviously show that the gene and protein regulatory networks display a protective network property just like the nested bow-tie architecture. Although there exist a lot of protein interactions, the existence of a conserved core and versatile weak linkages provides an opportunity to alter network connections and further trigger appropriate biological responses without seriously affecting other parts of the network in response to environmental stresses. This investigation provides us with in-depth insight into the nature of the protection system of yeast against a variety of environmental stresses.

## 4. Conclusion

In this study, based on microarray data, ChIP-chip data, and PPI data, gene/protein regulatory networks are constructed via dynamic model, ML estimation, AIC pruning methods, and RV to investigate their interplaying protection roles in response to environmental stresses on yeast. Then, a bow-tie core network structure is found to coordinate some functional pathways of these condensed gene/protein networks in response to a broader range of environmental stresses. The contributions of this study are pointed out as follows. (i) We construct two nonlinear stochastic coupled dynamic models to combine gene transcriptional regulatory networks and protein regulatory pathways (signaling pathway) in response to different stresses, that is, hypo-osmotic stress, sorbitol osmotic stress and pheromone stress (*α*-factor). (ii) We use the AIC method combined with backstepping selection procedure to prune the insignificant regulations in the stress-activated gene and protein regulatory networks. Furthermore we construct the ranking value of the significant regulations and interactions, *R* *V*, for each upstream protein (TF) to the corresponding target based on both the pruning result of the backstepping AIC and the identified parameters, ${\hat{b}}_{i,j}$ and ${\hat{d}}_{i,k}$. (iii) According to the significantly regulated proteins under each stress (Table 1), we find that some new found proteins probably participate in specific MAPK pathways in response to specific stress. (iv) According to functional patterns of the reconstructed stress-activated gene and protein regulatory networks, we find that the coordination of mutual interaction, feedforward loop, feedback activation, feedback inhibition, and cross talk by bow-tie core gene/protein network provides protection mechanisms in response to a broader range of environmental stress, that is, with speedy signaling transduction, noiseless and fast response, and network robustness under limited resources for preventing proliferation of inappropriate activation. Although the stress-activated gene and protein regulatory networks in response to different stresses are reconstructed by our models via microarray data, ChIP-chip data, and PPI data, a weakness of this study is that some ChIP-chip data is to date still unavailable for all 203 TFs to obtain whole protection mechanism for gene and protein regulatory networks in response to a broader range of environmental changes.

## 5. Methods

### 5.1. System Identification and AIC Backstepping Method

The coupled dynamic models in (1) and (2) can be combined and rewritten in the following matrix forms:

(4) $$Z_{i}=\Phi_{i}\Theta_{i}+\psi_{i},$$

where

(5) $$\begin{array}{cc} Z_{i} & ={\left[\begin{array}{cccccc} {\dot{X}}_{i}\left(t_{1}\right) & \cdots & {\dot{X}}_{i}\left(t_{N}\right) & {\dot{Y}}_{i}\left(t_{1}\right) & \cdots & {\dot{Y}}_{i}(t_{N}) \end{array}\right]}^{T},\\ \Phi_{i} & =\left[\begin{array}{cc} \begin{array}{ccccc} -X_{i}\left(t_{1}\right) & 1 & Y_{1}\left(t_{1}\right) & \cdots & Y_{J_{i}}\left(t_{1}\right)\\ \vdots & \vdots & \vdots & \ddots & \vdots \\ -X_{i}\left(t_{1N}\right) & 1 & Y_{1}\left(t_{N}\right) & \cdots & Y_{J_{i}}\left(t_{N}\right) \end{array} & 0\\ 0 & \begin{array}{cccccc} -Y_{i}\left(t_{1}\right) & 1 & X_{i}\left(t_{1}\right) & -Y_{i}\left(t_{1}\right)\cdot Y_{1}\left(t_{1}\right) & \cdots & -Y_{i}\left(t_{1}\right)\cdot Y_{K_{i}}\left(t_{1}\right)\\ \vdots & \vdots & \vdots & \vdots & \ddots & \vdots \\ -Y_{i}\left(t_{N}\right) & 1 & X_{i}\left(t_{1N}\right) & -Y_{i}\left(t_{N}\right)\cdot Y_{1}\left(t_{N}\right) & \cdots & -Y_{i}\left(t_{N}\right)\cdot Y_{K_{i}}\left(t_{N}\right) \end{array} \end{array}\right],\\ \Theta_{i} & ={\left[\begin{array}{cc} \underset{p_{i}}{\underset{︸}{a_{i}\;b_{i,0}\;b_{i,1}\;\cdots \;b_{i,j_{i}}}} & \underset{q_{i}}{\underset{︸}{\lambda_{i}\;d_{i,0}\;g_{i}\;d_{i,1}\;\cdots \;d_{i,K_{i}}}} \end{array}\right]}^{T}\in {ℜ}^{p_{i}+q_{i}},\\ \psi_{i} & ={\left[\begin{array}{cccccc} \varepsilon_{i}\left(t_{1}\right) & \cdots & \varepsilon_{i}\left(t_{N}\right) & \xi_{i}\left(t_{1}\right) & \cdots & \xi_{i}\left(t_{N}\right) \end{array}\right]}^{T},\;\text{where}\;\;p_{i}=J_{i}+2,\;\;q_{i}=K_{i}+3. \end{array}$$

Suppose that the noise components *ε*_*i*_ and *ξ*_*i*_ are independently and normally distributed, and the noise vector *ψ*_*i*_ has zero mean and variances *σ*_*i*_^2^ · *I* [70]. Using maximum likelihood (ML) method, we solve the parameter estimation problem with the optimum estimation ${\hat{\Theta }}_{i}$ and ${\hat{\sigma }}_{i}^{2}$. The likelihood function of *Z_i_* is defined as follows:

(6) $$\begin{array}{cc} & p_{i}\left(Z_{i}\;|\;\Theta_{i},{\sigma_{i}}^{2}\right)\\ & \;\;={\left(2\pi \right)}^{-1/2}\det\;{\left(\sigma_{i}^{2}\right)}^{-N/2}\\ & \;\;\;\times \exp\;\left[\left(-\frac{1}{2\sigma_{i}^{2}}\right)\cdot {\left(Z_{i}-\Phi_{i}\Theta_{i}\right)}^{T}\;\left(Z_{i}-\Phi_{i}\Theta_{i}\right)\right]. \end{array}$$

The log-likelihood function for the given *M* data points in *Z*_*i*_ can be defined as follows:

(7) $$\begin{array}{cc} L_{i}\left(\Theta_{i},{\sigma_{i}}^{2}\right) & =\text{constant}-\left(\frac{M}{2}\right)\ln\;\left[\det\;\left(\sigma_{i}^{2}\right)\right]\\ & \;-\left(\frac{1}{2\sigma_{i}^{2}}\right)\overset{M}{\underset{k=1}{\sum }}Z_{i}\left(t_{k}\right)-R_{\Phi_{i}}\left(t_{k}\right)\Theta_{i}, \end{array}$$

where *R*_Φ_*i*__ is the row vector of Φ_*i*_. We then estimate unknown parameters Θ_*i*_ and *σ*_*i*_^2^ by maximizing *L*_*i*_(Θ_*i*_, *σ*_*i*_^2^), that is, ∂*L*_*i*_(Θ_*i*_, *σ*_*i*_^2^)/∂Θ_*i*_ = 0 and ∂*L*_*i*_(Θ_*i*_, *σ*_*i*_^2^)/∂*σ*_*i*_ = 0, as follows:

(8) $$\begin{array}{c} \begin{array}{c} {\hat{\sigma }}_{i}^{2}=\frac{1}{M}{\left(Z_{i}-\Phi_{i}\Theta_{i}\right)}^{T}\left(Z_{i}-\Phi_{i}\Theta_{i}\right), \end{array}\\ \begin{array}{c} {\hat{\Theta }}_{i}={\left(\Phi_{i}^{T}\Phi_{i}\right)}^{-1}\Phi_{i}^{T}Z_{i}. \end{array} \end{array}$$

Because all the significant transcription regulations and PPIs do not simultaneously occur in each environmental condition, AIC combined with the backstepping method [71] is used to select the significant regulations, that is, *b*_*i*,*j*_ and *d*_*i*,*k*_, to prune the constructed networks to achieve the minimization of the AIC_*i*_, which is defined as follows:

(9) $$\text{AI}{\text{C}}_{i}\left(l\right)=\log\;\left[\frac{1}{M}{\left(Z_{i}-\Phi_{i}{\hat{\Theta }}_{i}\right)}^{T}\left(Z_{i}-\Phi_{i}{\hat{\Theta }}_{i}\right)\right]+\frac{2l}{M},$$

where *l* is the number of reserved parameters, that is, *p*_*i*_ + *q*_*i*_, in the backstepping AIC. The order of two parameters, that is, *b*_*i*,*j*_ and *d*_*i*,*k*_, in (4) are separately treated by the backstepping AIC. The order of the parameters selected by the backstepping AIC implies the importance of each PPI and transcription regulation, that is, the first selected protein (TF) is more important to the regulation of the target gene (or target protein) than the later selected proteins (TFs).

### 5.2. Ranking Value (RV)

Because both the order of the parameters selected by the backstepping AIC and the absolute values of the parameters imply the importance of each PPI and transcription regulation, we create a ranking value (RV) to evaluate the importance of each upstream protein (TF) to its target protein (gene). According to the identified models (3), we define indices to represent, respectively, the order of the absolute value of the parameters (*j*, *k*), that is, $\left|{\hat{b}}_{i,j}\right|<\left|{\hat{b}}_{i,j-1}\right|$ and $\left|{\hat{d}}_{i,j}\right|<\left|{\hat{d}}_{i,j-1}\right|,$ and the order of the parameters selected by the backstepping AIC (*m*, *n*); that is, ${\hat{b}}_{i,m}$ and ${\hat{d}}_{i,n}$ represent, respectively, the *m*th selected parameter and the *n*th selected parameter in the AIC selections. The ranking value (RV) is defined as RV = *j* · *n*/*j*_*i*_^′2^ and RV = *j* · *m*/*K*_*i*_^′2^, respectively, in the gene transcriptional regulatory network and the protein regulatory pathway; that is, the regulation ${\hat{b}}_{i,j}$ (or ${\hat{d}}_{i,k}$) with smaller RV implies that the regulation ${\hat{b}}_{i,j}$ (or ${\hat{d}}_{i,k}$) is more significant in the regulation of gene *i* (or protein *i*). 

### 5.3. *P*-Value Evaluation of Identified Regulation Relationships

We construct 1000 random permutations of *Y*_*j*_(*t*) in the first equation of (3) (or *Y*_*i*_(*t*)*Y*_*k*_(*t*) in the second equation of (3)) and then we use the random permutations to estimate 1000 different ${\hat{b}}_{i,j}$ (or ${\hat{d}}_{i,k}$) with AIC_*i*,*j*_^*b*^ (orAIC_*i*,*j*_^*d*^). The individual *P*-value for each ${\hat{b}}_{i,j}$ (or ${\hat{d}}_{i,k}$) is $P_{i,j}^{b}=\int_{\left|{\hat{b}}_{i,j}\right|}^{\infty }p_{\left|{\hat{b}}_{i,j}\right|}(x)dx\cdot \int_{-\infty }^{\text{AI}{\text{C}}_{i,j}^{b}}p_{\text{AI}{\text{C}}_{i,j}^{b}}(x)dx$ (or $P_{i,j}^{d}=\int_{\left|{\hat{d}}_{i,j}\right|}^{\infty }p_{\left|{\hat{d}}_{i,j}\right|}(x)dx\cdot \int_{-\infty }^{\text{AI}{\text{C}}_{i,j}^{d}}p_{\text{AI}{\text{C}}_{i,j}^{d}}(x)dx$) where $p_{\left|{\hat{b}}_{i,j}\right|}(x)$, *p*_AIC_*i*,*j*_^*b*^_(*x*), $p_{\left|{\hat{d}}_{i,j}\right|}(x),$ and *p*_AIC_*i*,*j*_^*d*^_(*x*) are, respectively, the probability distributions of $\left|{\hat{b}}_{i,j}\right|$, AIC_*i*,*j*_^*b*^, $\left|{\hat{d}}_{i,j}\right|$, and AIC_*i*,*j*_^*d*^. The *P*-values in the Supplementary tables are used to check the significance of the identified parameters, ${\hat{b}}_{i,j}$ and ${\hat{d}}_{i,k}$, in (3).

## Supplementary Material

The full tables of the gene and protein regulatory pathways under each stress combined with P-values and gene descriptions are available in Supplementary Material.

## Figures and Tables

**Table 1a:** (a) Hypo-osmotic stress

Target protein	Literature evidence	Upstream proteins within 40 preselected proteins											
SSK1^N^	[5–8]	SLN1 (0.050)	HOG1 (0.054)	STE50 (0.091)	CDC42 (0.095)	SSK2 (0.122)	STE20 (0.163)	OPY2 (0.181)	STE11 (0.272)	SHO1 (0.299)	PTP2 (0.560)	YPD1 (0.578)	SSK22 (0.905)
SSK2^N^	[4–8, 23]	STE11 (0.016)	SSK2 (0.018)	GPD1 (0.025)	PTP2 (0.041)	PTC1 (0.222)	YPD1 (0.249)	SSK1 (0.295)	FPS1 (0.531)	HOG1 (0.578)	SHO1 (0.819)		
PTP2^N^	[4, 5, 7, 8]	YPD1 (0.100)	SSK1 (0.140)	SSK2 (0.143)	SLN1 (0.160)	PTC1 (0.320)	STE20 (0.325)	HOG1 (0.330)	BCK1 (0.338)	PBS2 (0.500)			
STE50^P,O^	[6–8]	STE5 (0.047)	STE4 (0.208)	SSK1 (0.271)	SSK22 (0.286)	STE50 (0.306)	SSK2 (0.443)	SHO1 (0.479)	STE11 (0.627)				
MSB2^O^	[6]	CDC24 (0.015)	STE20 (0.028)	CLA4 (0.049)	CDC42 (0.056)	HOG1 (0.185)	SHO1 (0.432)	KSS1 (0.441)	STE12 (0.667)				
STE5^P^	[7]	CDC24 (0.045)	KSS1 (0.079)	STE7 (0.095)	STE50 (0.431)	STE20 (0.556)	STE12 (0.739)	STE2 (0.958)					
STE7^ P,O^	[6–8]	STE11 (0.035)	KSS1 (0.125)	STE5 (0.180)	STE7 (0.187)	STE4 (0.412)	STE12 (0.630)						
YPD1^N^	[6–8]	HOG1 (0.030)	SLN1 (0.060)	PTP2 (0.200)	PBS2 (0.200)	SSK2 (0.280)	SSK1 (0.640)						
AKR1^P^	[24]	KSS1 (0.014)	STE3 (0.236)	STE5 (0.267)	STE20 (0.365)	STE12 (0.391)	CLA4 (0.396)						
SLN1^N^	[5–8]	HOG1 (0.024)	PTC1 (0.111)	SSK1 (0.166)	PTP2 (0.291)	SHO1 (0.457)	YPD1 (0.571)						
PBS2^N^	[4–8, 23]	PKC1 (0.009)	RAS2 (0.067)	SSK22 (0.104)	HOG1 (0.124)	SHO1 (0.148)	PTP2 (0.260)						
MID2^I^	[7, 8]	SLT2 (0.080)	MID2 (0.125)	FPS1 (0.130)	PKC1 (0.158)	MKK1 (0.316)	BCK1 (0.656)						
MKK1^I^	[4, 7, 8]	PKC1 (0.004)	MID2 (0.045)	PTP2 (0.221)	RHO1 (0.238)	SLT2 (0.416)	BCK1 (0.544)						

**Table 1b:** (b) Sorbitol osmotic stress

Target protein	Literature evidence	Upstream proteins within 40 preselected proteins												
STE4^P^	[7]	STE12 (0.001)	TEC1 (0.005)	PKC1 (0.006)	HOG1 (0.014)	STE50 (0.015)	RHO1 (0.017)	STE11 (0.020)	STE7 (0.021)	STE20 (0.023)	CDC24 (0.024)	STE2 (0.037)	STE5 (0.209)	STE3 (0.236)
SHO1^O^	[4–8, 23]	SHO1 (0.001)	SSK1 (0.002)	MSB2 (0.003)	HOG1 (0.004)	SSK2 (0.006)	STE11 (0.007)	PKC1 (0.008)	STE50 (0.013)	STE20 (0.014)	PBS2 (0.017)	SLN1 (0.024)		
STE11^P,O^	[4–8, 23]	TEC1 (0.003)	STE20 (0.004)	KSS1 (0.007)	SSK1 (0.010)	STE7 (0.011)	STE11 (0.014)	CLA4 (0.014)	SHO1 (0.016)	GPD1 (0.017)	STE4 (0.025)	STE5 (0.516)		
PTP2^N^	[4, 5, 7, 8]	SSK2 (0.020)	PTC1 (0.045)	HOG1 (0.180)	PTP3 (0.195)	BCK1 (0.200)	SSK1 (0.213)	SLT2 (0.360)	STE20 (0.405)	PBS2 (0.413)	SLN1 (0.500)	YPD1 (0.525)		
STE50^P,O^	[6–8, 23]	STE50 (0.022)	SSK1 (0.074)	CDC42 (0.095)	SSK2 (0.111)	STE4 (0.118)	STE5 (0.231)	SSK22 (0.249)	STE11 (0.281)	SHO1 (0.391)	OPY2 (0.408)			
STE5^P^	[7]	KSS1 (0.003)	CDC24 (0.011)	STE11 (0.013)	HOG1 (0.024)	STE7 (0.026)	STE12 (0.030)	STE50 (0.031)	STE5 (0.073)	STE4 (0.333)	STE2 (0.809)			
PBS2^N^	[4–8, 23]	KSS1 (0.002)	RAS2 (0.004)	STE11 (0.009)	PKC1 (0.010)	PTP2 (0.011)	YPD1 (0.012)	SSK22 (0.017)	HOG1 (0.019)	PTC1 (0.023)	SHO1 (0.026)			
SSK2^N^	[4–8, 23]	SSK2 (0.014)	STE50 (0.058)	GPD1 (0.080)	FPS1 (0.086)	HOG1 (0.144)	PTC1 (0.154)	PTP2 (0.168)	YPD1 (0.229)	SSK1 (0.288)	STE11 (0.736)			
SSK1^N^	[4–8, 23]	HOG1 (0.061)	PTP2 (0.092)	STE50 (0.092)	YPD1 (0.112)	SSK22 (0.163)	SSK2 (0.255)	STE11 (0.357)	SHO1 (0.643)	STE20 (0.796)				
STE12^ P,O^	[7, 8]	STE7 (0.002)	STE11 (0.004)	STE20 (0.006)	MSB2 (0.012)	RAS2 (0.014)	STE5 (0.289)	STE4 (0.351)	STE2 (0.907)					
FPS1^N^	[5]	STE11 (0.001)	GPD1 (0.003)	HOG1 (0.007)	MID2 (0.009)	SSK22 (0.011)	FPS1 (0.014)	SSK2 (0.016)	SLT2 (0.017)					
MSB2^N^	[6]	HOG1 (0.048)	CDC42 (0.097)	STE12 (0.104)	CDC24 (0.135)	CLA4 (0.228)	KSS1 (0.249)	STE20 (0.824)						
FAR1^P^	[25]	STE11 (0.007)	STE12 (0.011)	CDC42 (0.021)	CDC24 (0.022)	SSK2 (0.023)	STE5 (0.086)	STE4 (0.296)						
SPA2^I^	[7]	SLT2 (0.001)	SSK2 (0.005)	STE7 (0.010)	CLA4 (0.012)	STE11 (0.015)	RAS2 (0.016)	STE12 (0.022)						
PTC1^N^	[4, 5, 7, 23]	CLA4 (0.001)	SSK2 (0.002)	PBS2 (0.004)	PKC1 (0.007)	PTP2 (0.010)	SLN1 (0.015)							
NBP2^N^	[23]	SSK2 (0.025)	PTC1 (0.044)	BCK1 (0.127)	PTP2 (0.222)	STE20 (0.452)	SLT2 (0.645)							
GIC2^P,O^	[26]	MSB2 (0.180)	STE50 (0.413)	STE20 (0.471)	CDC24 (0.645)	CLA4 (0.676)	CDC42 (0.712)							
OCH1^O^	[7]	KSS1 (0.050)	CLA4 (0.223)	STE20 (0.331)	STE7 (0.331)	STE12 (0.347)	STE11 (0.744)							
GPA1^P^	[7, 27]	STE7 (0.051)	STE5 (0.112)	STE2 (0.147)	STE4 (0.357)	STE12 (0.388)	STE11 (0.520)							
SKN7^N^	[4, 7]	PKC1 (0.005)	YPD1 (0.009)	CDC42 (0.009)	SLN1 (0.011)	PTC1 (0.014)	RHO1 (0.016)							
SST2^P^	[28]	STE50 (0.001)	KSS1 (0.015)	SHO1 (0.016)	STE4 (0.175)	STE5 (0.242)	STE2 (0.623)							
WSC3^I^	[29]	RHO1 (0.032)	SSK22 (0.036)	STE11 (0.041)	PKC1 (0.100)	SSK2 (0.215)	MID2 (0.299)							
SKM1^O^	[4]	CLA4 (0.001)	SWI6 (0.010)	STE20 (0.015)	CDC42 (0.020)	PTC1 (0.026)	STE3 (0.051)							
RGA1^O^	[30]	CLA4 (0.001)	RHO1 (0.006)	CDC42 (0.007)	STE20 (0.019)	PBS2 (0.032)	STE4 (0.442)							
MKK1^I^	[4, 7, 8]	PKC1 (0.028)	RHO1 (0.038)	BCK1 (0.061)	SLT2 (0.153)	MID2 (0.436)	PTP2 (0.670)							
BEM4^I^	[31]	CLA4 (0.006)	HKR1 (0.011)	CDC24 (0.016)	CDC42 (0.019)	STE20 (0.021)	RHO1 (0.025)							

**Table 1c:** (c) Pheromone stress

Target Protein	Literature evidence	Upstream proteins within 40 preselected proteins												
STE20^P,O^	[6–8, 23]	STE4 (0.001)	STE3 (0.002)	STE5 (0.008)	CLA4 (0.010)	MSB2 (0.016)	SSK1 (0.016)	STE12 (0.069)	RAS2 (0.080)	CDC24 (0.175)	PTP2 (0.342)	SLT2 (0.471)	CDC42 (0.535)	SHO1 (0.634)
HOG1^N^	[4–8, 23]	STE5 (0.001)	STE4 (0.002)	SSK2 (0.035)	YPD1 (0.099)	MSB2 (0.109)	PTP2 (0.202)	SSK1 (0.235)	FPS1 (0.265)	SHO1 (0.385)	PTC1 (0.393)	SLN1 (0.417)	PTP3 (0.420)	STE11 (0.718)
STE4^P^	[7]	STE5 (0.012)	STE3 (0.019)	STE2 (0.022)	RHO1 (0.036)	STE50 (0.126)	PKC1 (0.128)	STE7 (0.201)	KSS1 (0.231)	STE20 (0.261)	HOG1 (0.365)	STE12 (0.370)	STE11 (0.373)	CDC24 (0.544)
STE50^P,O^	[6–8, 23]	SSK1 (0.011)	STE50 (0.023)	STE5 (0.106)	OPY2 (0.251)	SSK22 (0.318)	SHO1 (0.321)	SSK2 (0.416)	CDC42 (0.539)	STE4 (0.739)	STE11 (0.749)			
SHO1^O^	[4–8, 23]	PBS2 (0.029)	SSK1 (0.102)	STE11 (0.132)	MSB2 (0.165)	STE50 (0.217)	HKR1 (0.260)	SSK2 (0.263)	HOG1 (0.607)	SHO1 (0.640)	STE20 (0.725)			
SPA2^I^	[7]	MKK1 (0.070)	STE7 (0.085)	PKC1 (0.162)	CLA4 (0.188)	RAS2 (0.234)	STE11 (0.304)	SSK2 (0.444)	RHO1 (0.609)	SLT2 (0.641)	STE12 (0.672)			
PKC1^I^	[4, 7, 8]	STE4 (0.010)	PBS2 (0.063)	SLT2 (0.080)	SHO1 (0.187)	BCK1 (0.313)	RHO1 (0.340)	MKK1 (0.365)	PTC1 (0.644)	MID2 (0.819)				
BEM1^P^	[4, 24, 32]	STE4 (0.004)	RHO1 (0.009)	STE5 (0.009)	CDC42 (0.033)	CDC24 (0.037)	STE7 (0.045)	CLA4 (0.065)	STE20 (0.245)	STE11 (0.411)				
STE5^P^	[7]	STE5 (0.012)	STE4 (0.060)	STE12 (0.151)	HOG1 (0.173)	STE11 (0.230)	CDC24 (0.235)	STE2 (0.303)	STE50 (0.346)	KSS1 (0.560)				
STE12^P,O^	[7, 8]	STE4 (0.012)	STE5 (0.016)	STE7 (0.076)	STE12 (0.078)	RAS2 (0.109)	TEC1 (0.180)	STE20 (0.217)	MSB2 (0.431)	STE11 (0.657)				
PBS2^N^	[4–8, 23]	SHO1 (0.003)	YPD1 (0.005)	RAS2 (0.010)	PTC1 (0.011)	PKC1 (0.021)	KSS1 (0.022)	SSK2 (0.025)	HOG1 (0.026)	SSK22 (0.029)				
SSK2^N^	[4–8, 23]	SSK2 (0.009)	SSK1 (0.034)	PBS2 (0.106)	HOG1 (0.106)	STE50 (0.227)	SHO1 (0.386)	YPD1 (0.431)	FPS1 (0.484)	PTC1 (0.567)				
PTP2^N^	[4, 5, 7, 8]	PBS2 (0.039)	MKK1 (0.039)	HOG1 (0.047)	BCK1 (0.150)	PTP3 (0.249)	SSK2 (0.266)	PTC1 (0.288)	SSK1 (0.305)	SLT2 (0.798)				
CLN2^P^	[4, 7, 33, 34]	SWI6 (0.001)	STE4 (0.001)	STE11 (0.003)	CLA4 (0.007)	SLT2 (0.009)	CDC42 (0.074)	STE20 (0.096)	RAS2 (0.525)	PKC1 (0.552)				
NBP2^N^	[23]	PTP2 (0.021)	STE20 (0.065)	PBS2 (0.120)	HOG1 (0.477)	SSK2 (0.529)	SLT2 (0.567)	BCK1 (0.645)	PTC1 (0.696)					
BCK1^I^	[4, 7, 8]	HKR1 (0.003)	MKK1 (0.065)	PTP2 (0.139)	BCK1 (0.164)	SLT2 (0.166)	PKC1 (0.250)	CLA4 (0.267)	MID2 (0.911)					
KSS1^P,O^	[6–8]	STE4 (0.022)	STE12 (0.031)	HOG1 (0.179)	STE7 (0.190)	PBS2 (0.237)	TEC1 (0.305)	MSB2 (0.692)						
SMI1^I^	[35, 36]	SLT2 (0.029)	PKC1 (0.236)	CLA4 (0.251)	FPS1 (0.340)	BCK1 (0.380)	PTC1 (0.650)	MID2 (0.790)						
FPS1^N^	[5]	MID2 (0.028)	STE11 (0.047)	SSK22 (0.053)	SSK2 (0.063)	GPD1 (0.202)	SLT2 (0.260)	HOG1 (0.709)						
BNI1^I^	[7, 37, 38]	RHO1 (0.000)	CDC42 (0.000)	STE11 (0.004)	CLA4 (0.005)	STE12 (0.006)	PKC1 (0.009)	BCK1 (0.011)						
CLA4^O^	[5, 6]	MSB2 (0.001)	CDC24 (0.002)	SLT2 (0.003)	BCK1 (0.003)	STE20 (0.006)	STE11 (0.006)	CDC42 (0.007)						
PTC1^N^	[4, 5, 7, 23]	SSK2 (0.070)	PKC1 (0.107)	PTP2 (0.172)	PBS2 (0.342)	HOG1 (0.680)	SLT2 (0.736)							
STE7^P,O^	[6–8]	STE5 (0.012)	STE4 (0.089)	STE12 (0.130)	STE7 (0.331)	STE11 (0.385)	KSS1 (0.568)							
SLT2^I^	[4, 7, 8]	BCK1 (0.001)	PKC1 (0.001)	MID2 (0.003)	FPS1 (0.003)	PTC1 (0.006)	CLA4 (0.007)							
FAR1^P^	[25]	SSK2 (0.008)	STE12 (0.010)	STE5 (0.011)	CDC42 (0.021)	CDC24 (0.029)	STE4 (0.035)							
MID2^I^	[7, 8]	SLT2 (0.019)	MID2 (0.019)	FPS1 (0.065)	MKK1 (0.083)	PKC1 (0.150)	RHO1 (0.166)							
FKS1^I^	[4, 7]	MKK1 (0.009)	SLT2 (0.030)	MID2 (0.050)	RHO1 (0.191)	PKC1 (0.332)	PTC1 (0.414)							
SKM1^O^	[4]	STE3 (0.027)	PTC1 (0.125)	CLA4 (0.131)	SWI6 (0.150)	STE20 (0.197)	CDC42 (0.312)							
MKK1^I^	[4, 7, 8]	BCK1 (0.022)	PTP2 (0.104)	SLT2 (0.196)	PKC1 (0.329)	RHO1 (0.678)	MID2 (0.726)							
BEM4^I^	[31]	PKC1 (0.009)	RHO1 (0.015)	CDC24 (0.018)	CDC42 (0.020)	CLA4 (0.024)	HKR1 (0.027)							
